# Enhanced composting as a way to a climate-friendly management of coffee by-products

**DOI:** 10.1007/s11356-020-08742-z

**Published:** 2020-04-18

**Authors:** Macarena San Martin Ruiz, Martin Reiser, Martin Kranert

**Affiliations:** Institute for Sanitary Engineering, Water Quality and Solid Waste Management, University of Stuttgart, Bandtäle 2, 70569 Stuttgart, Germany

**Keywords:** Composting, Coffee by-products, Coffee pulp, Methane, Emission rates, Greenhouse gases

## Abstract

This study investigated the performance of aerobic windrow systems by using coffee by-products and green waste to reduce gaseous emissions. Thereafter, a comparison with the current treatment and gaseous emissions at a Coffee Mill in Costa Rica was made. Two different studies where performed in Germany (pile I and II) and one study in a Coffee Mill in Costa Rica (pile III). Temperature, water content, and pH were the key parameters controlled over 35 days in all the systems. Moreover, CH_4_ emission rates were quantified by a FTIR and by a portable gas detector device where the emissions reached values 100 times higher when coffee by-products as a unique material for the composting process was used. Results show that highest emission rates during the composting process for pile I was 0.007 g(m^2^)^−1^ h^−1^, for pile II 0.006 g(m^2^)^−1^ h^−1^, and for pile III 3.1 g(m^2^)^−1^ h^−1^. It was found that CH_4_ emissions could be avoided if the mixture and the formation of the windrow piles were performed following the key parameter for composting, and the usage of additional material is used. With this, the reduction of CH_4_ emissions at the Mill in Costa Rica could be achieved in the future.

## Introduction

Composting among the years has become a promising natural way of recycling organic matter and producing fertilizer under low operating costs and minimal technology (Haug 1993; Misra et al. 2003). It is defined as “a biological decomposition and stabilization of organic substrates, under conditions that allow the development of thermophilic temperatures as a result of biologically produced heat, to produce a final product that is stable, free of pathogens and plant seed and can be beneficially applied to land*”* (Artola et al. 2015; Misra et al. 2003)*.* The base of composting is not the complete decomposition of the input components but rather to prepare a biologically stable material which is not exposed to a process of rapid decomposition or undesirable rotting (Burg et al. 2011). During the process, temperature has been one key factor in composting which has been used as a tool to follow the degree of stabilization as a result of microbial activities during the process (Bueno et al. 2007). One of the disadvantages of composting is the formation of greenhouse gases (GHG) such as methane (CH_4_) that enhance the global warming (Zhu-Barker et al. 2017). The GHG formation occurs from the activity of microorganisms during the composting process (Sun et al. 2014). CH_4_ corresponds to the main product when the windrow piles do not receive the necessary oxygen at the core of the windrow (Amlinger et al. 2008). In the agricultural sector, GHG represents 24% of the total emissions globally, excluding carbon dioxide (CO_2_) since the gas generated is climate-neutral carbon, for the reason that it originate from the conversion of organic material and dead organic matter (Amlinger et al. 2008; Sun et al. 2014).

**Fig. 1:**
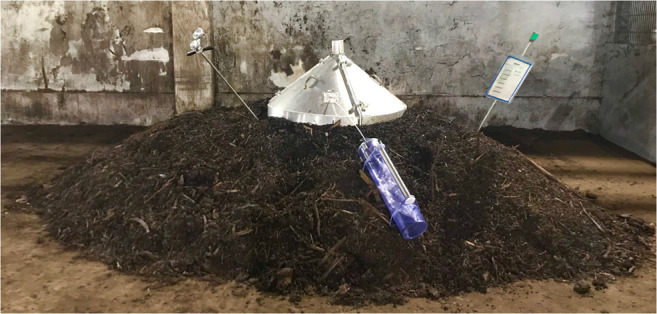
Gas measurement at the composting plant facility

Currently, the Mill of study in Costa Rica is treating its coffee residue to produce compost, where the main material is based on coffee husk and coffee pulp (Fig. 1) as coffee by-products (Zarrinbakhsh et al. 2016).

During the wet process of coffee bean extraction, these coffee by-products are divided into coffee husk, skin, pulp, mucilage, and parchment (Esquivel and Jiménez 2012; Iriondo-DeHond et al. 2019). The main coffee by-product obtained during wet or semi-dry processing is the coffee pulp, which corresponds to approximately 29% on a dry-weight basis (Blinová et al. 2017; Heeger et al. 2017), where one ton of coffee pulp is generated for every two tons of green coffee produced (Esquivel and Jiménez 2012). Coffee pulp is an organic waste that contributes to pollution and environmental problems when the coffee berries are ripe and processed during the wet method (Lardé 1989; Blinová et al. 2017). In each harvest at this Mill in Costa Rica, where wet process is the main method to process the coffee cherries, the coffee by-products produced are approximately 37,000 Mg/year.

Understanding greenhouse gas emissions formation is an important criterion in future evaluation options for climate change mitigation within the coffee sector (Rahn et al. 2014; Nieters et al. 2015). Coffee by-products are also the contributors to climate change as a result of their greenhouse gases emitted (Rahn et al. 2014); therefore, their emissions play an important role.

In this study, first a characterization of coffee by-products and green waste products was completed. Based on this, it was proposed to carry out a pilot plan in Germany with different green waste materials and coffee pulp to investigate the behavior of this coffee by-product during the composting process for 35 days. In addition, to determine the capacity to reduce greenhouse gases emissions and other harmful impacts on the environment, a comparison with the current composting process at the Mill in Costa Rica and the composting process which was performed in Germany were made.

Finally, the quantification of CH_4_ emission rates in three different piles containing coffee pulp as a main component was performed to analyze the relevance of external materials and their relationship with the current CH_4_ emissions within the usage of coffee pulp during composting.

## Materials and methods

### Windrows description

Composting profile, chemistry, and greenhouse gas emissions were monitored at the composting plant facility in Germany and at the Mill in Costa Rica. The coffee pulp was sundried at the Mill and shipped to Germany. Once it arrived in Germany, naturally the material was humidified until the fresh percentage that the fruit possess. On the other hand, the green waste for the experimental site in Germany was obtained from the composting plant facility where the windrows were built. Three windrow piles were monitored in total, and the main component was the coffee by-product.

The first pile (I) was formed using 50% based on volume of coffee pulp, a mixture of 50% based on volume of green waste, and a structural material at the composting plant facility during the winter season. Pile II was formed using 20% more based on volume of coffee pulp than in pile I, a mixture of 30% based on volume of green waste, a and structural material composted during the spring season, whereas pile III was completely made with coffee by-products during the summer season using the current methodology of the mill at the Mill in Costa Rica. Two windrows piles (I and II) were monitored in Germany and afterwards compared with the current emissions obtained from a previous study (San Martin Ruiz et al. 2018).

The piles were running for a period of 35 days, taking into account the specifications at the composting plant facility to produce compost using green waste material as an input. The material was turned weekly using a Tracturn® windrow turner at the composting plant facility and in Costa Rica with a Backhus® windrow turner. The sizes of the piles in Germany were approximately 6 m^3,^ while in Costa Rica, the pile was approximately 90 m^3^ with 1.2 m high and 2.2 m wide for all the piles.

Gas samples were collected weekly before each turning event. Compost samples were collected before and after each turning event. Temperature was measured continuously using an Armatherm thermometer® T-logger in pile I and II. On the other hand, at the Mill, the temperature was measured twice a day during the mornings and afternoons using a compost thermometer and the samples for the key parameters where collected weekly.

### Compost sampling and analyses

Compost input material which was used to form the windrow piles in Germany were sampled to quantify the gravimetric water content (WC), pH, electrical conductivity (*κ*), bulk density (Ƿ_t_), dry solids (DS), volatile solids (VS), and carbon to nitrogen (C/N) ratio which are shown in Table 1. Once the windrow piles were formed, WC, pH, and C/N ratio were performed weekly. The samples were collected before and after each turning event and were taken from 5 different locations and depths along the pile to obtain a representative sampling over the entire pile. Water content was calculated from field moist and oven-dry (105 °C for 48–72 h) mass of compost according to the DIN EN 13040 (BGK 2017). The pH was extracted from 20 g (wet weight) of compost with 180 mL of CaCl_2_ and assessed by potentiometric measurements.

**Table 1:** Constituents of input material for each windrow pile

Windrow system	Material	C/N	pH	*κ* mscm^−1^	WC %	DS %	Ƿ_t_ g/L	VS %
Input material Germany								
I and II	Humidified coffee pulp	20 ± 0.2	6 ± 0.1	1.1 ± 0.1	79.7 ± 0.2	20.3 ± 0.2	564 ± 6.0	90.9 ± 0.4
I	Green waste^1^	22 ± 0.1	5 ± 0.1	2.0 ± 0.4	21.3 ± 0.2	78.7 ± 0.3	505 ± 5.0	98.1 ± 0.1
I	Structure material^2^	28 ± 0.3	6 ± 0.1	0.9 ± 0.1	25.8 ± 0.2	74.2 ± 0.1	232 ± 3.0	67.8 ± 1.3
II	Structure material^3^	67 ± 0.5	6 ± 0.1	0.4 ± 0.3	31.8 ± 1.2	68.2 ± 0.8	190 ± 5.0	77.2 ± 0.1
II	Green waste^4^	31 ± 0.1	7 ± 0.2	0.7 ± 0.2	35.8 ± 0.0	64.2 ± 0.5	550 ± 5.0	53.6 ± 0.2
Input material in Costa Rica								
III	Fresh pulp	13.4 ± 0.2	3.9 ± 0.1	1.6 ± 0.1	84.9 ± 0.2	15.1 ± 0.7	600 ± 10.0	88.9 ± 1.8

^1,2:^Green waste and structural material during winter season in Germany

^3,4^Green waste and structural material during spring season in Germany

Standard deviation of the mean values *n* = 3 for all the values

Electricity conductivity was extracted from 20 g (wet weight) of compost with 180 mL of distillated water. Volatile solids were performed and calculated according to the Federal Compost Quality Assurance Organization (FCQAO) (Bidlingmaier 2003) and according to the DIN 18128. C/N ratios were performed using a vario Max CN element analyzer GmbH® following the DIN ISO 10694.

For the VS, three replicates of 10 g were inserted into a porcelain crucible with known weight. The samples were inserted into a furnace at 550 °C and burned until constant weight according to the DIN 18128. Thereafter, the volatile solids were calculated for each replicate, and the average of the three values was taken to represent the organic content of the sample.

### Gas measurements and collection

At the composting plant facility in Germany, an open upper part chamber was placed on top of the windrow piles and inserted approximately 5–10 cm deep into the windrow to seal the chamber against atmospheric influences in order to quantify the windrow emissions focusing on CH_4_ measurements. At each sampling event, the sample was taken from at the top of the windrow pile since the main emissions are emitted at this area of the pile (Ahn et al. 2011).

During sampling, the flow principle passing through the sampling hood at a passive area source was used to extract a defined amount of air (open upper part chamber), covering the entire area required for sampling as a function of the constant flow of emissions and supply of ambient air (Bidlingmaier 2003; VDI 3475 part 2 2005). The sampling device consists of a vacuum vessel which is discharged by using a vacuum pump, and at the same time, a hose is connected between the open upper part chamber and the vacuum vessel to collect the gas.

**Fig. 2:**
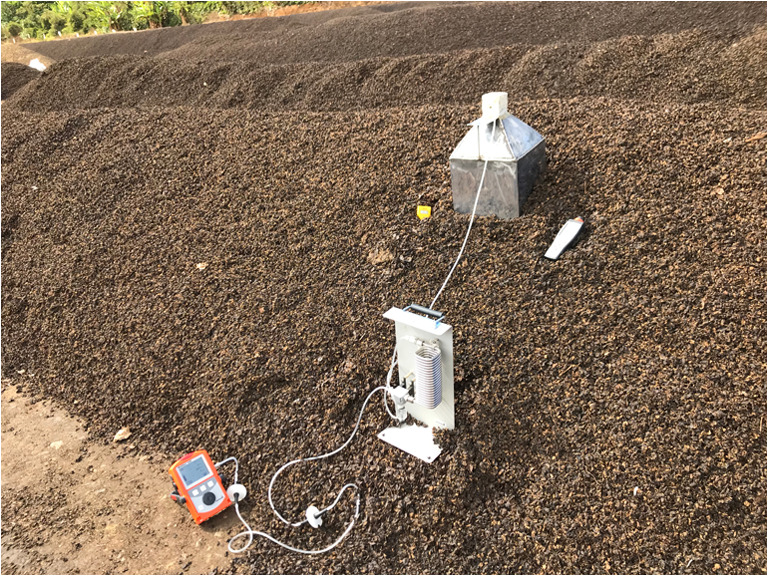
Gas measurement at the Mill in Costa Rica

When the sampling device begins pumping, the sampling bag, made of Nalophan, absorbs the inner gas (Fig. 1). The upper part was uncovered allowing the ambient air to enter and to be mixed inside of the sampling hood. The gas collection was done weekly for a period of 30 min in order to collect 6 L of gas in a sampling bag. The gas samples were analyzed using Fourier-transform infrared spectroscopy (FTIR). Thereafter, the gas measurements were compared according to the previous study performed at the Mill in Costa Rica, where an open upper part sampling hood was used to measure the gas concentration by using a portable gas detector device (Fig. 2) (San Martin Ruiz et al. 2018).

### Emission rates

The emission rates were calculated according to the sampling chamber volume, sampling chamber area, flow volume of the measurement equipment, and a specific flow rate each measurement performed. The following equations describe the calculations and formulas used to obtain the emission rates (Clauß et al. 2019; San Martin Ruiz et al. 2018).

1 $$ {\mathrm{C}}_{\mathrm{C}\mathrm{H}4}=\left({\mathrm{M}}_{\mathrm{C}\mathrm{H}4\ast }\ {\upvarphi}_{\mathrm{C}\mathrm{H}4}\right)/{\mathrm{V}}_{\mathrm{mo}}\mathrm{l} $$

C_CH4_: methane concentration, (mg/m^3^)

M_CH4_: molar mass of methane, (g/mol)

V_mo_l: 22.4139 L at standard conditions

φ_CH4_: methane in volume percentage or in ppm.

For Emission rates:

2 $$ {\mathrm{q}}_{\mathrm{C}\mathrm{H}4}=\left({\mathrm{C}}_{\mathrm{C}\mathrm{H}4}\ast {\mathrm{V}}_{\mathrm{gas}}\right)/{\mathrm{A}}_{\mathrm{H}} $$

q_CH4_: emission rate of methane, (g/m^2^ h)

C_CH4_: methane concentration, (mg/m^3^)

A_H_: hood area, (m^2^)

V_gas_: gas flow volume, (L/h)

### Statistical analysis

In total 25 gas measurements during morning and afternoon were performed at the mill in Costa Rica, obtaining up to 5 replicas among the pile. The data for pile III was subjected to one-way analysis of variance ANOVA for Windows. Significant level of *p* ≤ 0.001 for pile III was used for all mean values. Meanwhile for piles I and II, measurements were performed once per week. Nevertheless, a 2% linearity deviation was considered for the results.

## Results

**Fig. 3:**
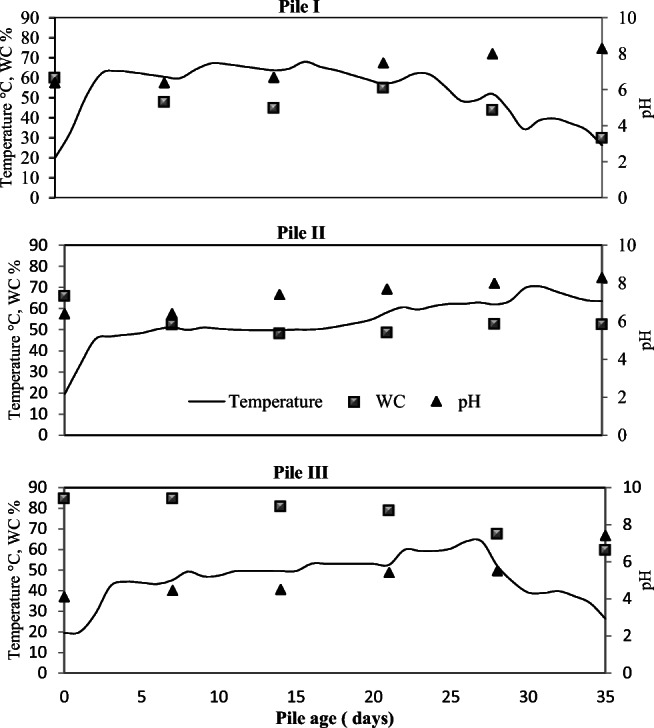
Temperature, water content concentration, and pH in three piles

Figure 3 shows a summary of the input features of the compost material measured when the windrows were built. All three piles were having triangular shape, width, and height where the only difference was the length of the pile, and this was due to the viability of coffee pulp material shipped to Germany. Therefore, pile I and II were shorter than at the Mill in Costa Rica. After receiving the dehydrated coffee pulp, certain analyses were carried out to estimate the amount of water necessary to humidify the pulp and with this, to be able to carry out the simulation of fresh material at the composting plant facility in order to follow closely and in a real sense how the coffee by-products are handled at the mill in Costa Rica. As can be seen in Table 1, the hydrated material obtained a difference of 5 percentage point with respect to the percentage of original moisture. C/N ratio have increased during the humidification of the coffee pulp, which indicates that during the drying process, the material had a nitrogen transformation; therefore, the C/N ratio increased as well as its pH (Hao and Benke 2008). All the previous analysis indicates that the preliminary results are significant for the study and a comparison between the systems can be made.

### Parameters and temperatures profiles in composting windrows

All the temperature data from the T-logger was given weekly from the personnel of the composting plant facility whereas in Costa Rica, the temperature was measured twice per day (mornings and afternoons). The composting process was performed during different times of the year; therefore, the feedstocks of each pile were based on the available material that the composting plant facility had during that season in addition to the piles experiencing different seasonal conditions. Pile I experienced a winter climate with an average ambient temperature of 5.4 °C. Pile II experience a spring climate season between 13 and 18 °C. Pile III accomplished a summer climate at the Mill in Costa Rica, where the range of ambient temperature was 28 °C without any rain event. Significant temperature increases were observed between the windrow systems. Pile I and II were performed in a place covered by a roof, while pile III was performed in an open field at the Mill. Generally, the temperature was higher where a high percentage of green waste was added, reaching rapidly 60 °C within the first week even if the windrow system was performed during winter season and under low ambient temperature. Figure 3 shows that WC decreases among the pile age of the windrow systems I and II, whereas in pile III occurred no variation during 20 days of pile age, and the high value is attributed to the high WC of the coffee by-product. It is important to control the degree of degradation during the composting process, because this parameter is used to give information regarding the decomposition process (Burg et al. 2011). Compost microorganisms work best under neutral rather than acidic conditions (Sundberg 2008), where at pH levels under 5, the microorganisms’ inhibition can be noticed (Bachert and Wattanachira 2008; Sundberg et al. 2013). Organic acids are neutralized within the process, and mature compost generally has a pH between 6 and 8 (Burg et al. 2011; Sundberg 2008). The pH increased in pile I and II over the pile age, while pile III remained acidic. Pile I and II where carried out under a C/N ratio of 25:1 and 30:1, respectively. The WC in pile I had a rapid evaporation or absorption within the material during this process. No water was added into pile I in order to maintain the humidity from the pile itself. In pile II, the WC was between 30 and 60% during the pile age, where incorporation of small amounts of water was needed to maintain a WC between 40 and 55% until the sanitation process occurred. Pile III maintained moisture between 40 and 66% itself during the total process, and no additional water was added during the process in 35 days.

### Methane emissions from compost windrow piles

**Fig. 4:**
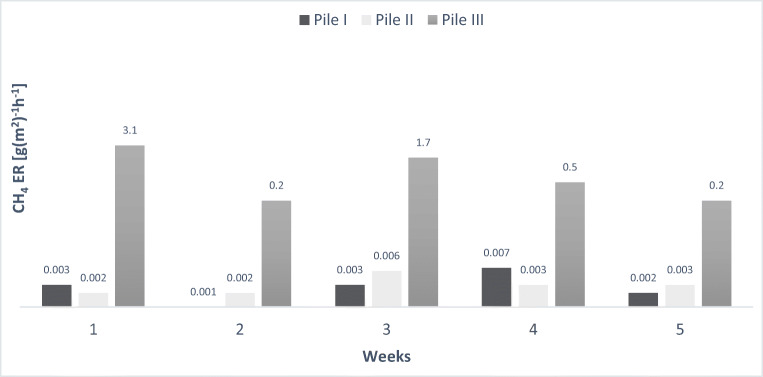
CH_4_ emission rates (ER) [g(m^2^)^−1^ h^−1^] from the windrow piles plotted with the logarithmic scale to the number of weeks of the process

All the CH_4_ emissions were measured before each turning event at the pile in order to achieve the similar conditions for a congruent comparison between the systems. During the conversion of methane concentration to emissions rates (ER), the flow through the upper part chamber was considered. Figure 4 shows the results of emissions rates over the pile age represented in weeks for the three different windrow systems. The emission rates in pile I and II were reduced drastically in comparison with pile III.

However, the pile II had obtained an increase of CH_4_ emissions in comparison with pile I. This can be related to the amount of coffee pulp increased a 20%. The maximum values of CH_4_ emission rates in pile I were found during the fourth week, pile II during the third week, and pile III at the first week of composting which can be related to the poor aeration and high WC levels (85% of WC reported during the first week in pile I), enhancing the methane formation (Amlinger et al. 2008; Hrad et al. 2014; Jenkins 2011; VDI 3475 part 3 2006).

## Discussions

Firstly, the attention was focused on the fact that the amount of green waste and coffee by-product for the composting could be comparable to each other in terms of gaseous emissions and that the additional material would be easily accessible and collected. Therefore, the selection of measurement methods had to allow comparable data in the various types of treatments, as well as CH_4_ emissions. Temperature profiles show that after increasing the addition of coffee pulp into the windrow pile, the temperature will take up to 25 days to reach the sanitation peak (Diaz et al. 2007; Jenkins 2011).

On the other hand, the temperature at any point depends mainly on the amount of heat produced by microorganisms and the amount lost through aeration and surface cooling. Therefore, the time where the system remains with high temperatures will depend on the chemical composition of the ingredients, as well as the volume of the system. During the thermophilic stage (40–60 °C), the degradation occurs faster and can take from days to months depending on the material and the composition of the ingredients (Cornell University 2001; Sierra et al. 2017; VDI 3475 part 2 2005). This stage of composting contains a relevant path in order to destroy germs that are sensitive to the temperature (Msunar 2009; Sierra et al. 2017; VDI 3575 part 1 2003). In pile I and II, the thermophilic stage starts during the first 2 days of composting, while pile III needed more days to achieve the mesophilic phase. During the first phase of the process, the pH value tends to drop due to liberation of organic acids.

Thereafter, once the process is moved to the next phases, the pH value tends to rise since all the organic acids are broken down as well as the alkaline effect of the inorganic salts which tend to be bonded to the organic material. At the end of the phases, the pH value must fluctuate in the neutral to basic range (Kranert 2017; VDI 3575 part 1 2003).

During the composting process, high temperatures in the windrow pile kill worm eggs and pathogens resulting in a compost sanitation (Bidlingmaier 2003; Federal Compost Quality Assurance Organization 1994; VDI 3475 part 2 2005) which were reached by pile I during the first days of composting whereas in pile II and III after 20 days of composting time. An optimal C/N ratio for the development of microorganisms and bacteria responsible for composting is between 25:1 and 40:1 (Ahn et al. 2011). Based on this, the piles at the composting plant facility followed the appropriated ranges of C/N during the process.

Three important factors that affect the temperature change are the WC, sufficient oxygen in the windrow pile, and the shape of the pile. The pore-volume relationship during the process is an important prerequisite in order to enhance a good composting process. If the material does not possess enough oxygen and high amount of water content, the low air pore volume in the pile is being affected, and therefore, a release odor due to anaerobic metabolites occur. Triangular shape results in a larger surface-to-volume ratio, giving a natural convection allowing adequate aeration (VDI 3575 part 1 2003). Giving an adequate moisture level, which was given in pile I and II, the microorganism activity is maintained for a longer period. Low water content in the decomposition material may partially or completely inhibit the activity and reproduction rate of the microorganisms (dry stabilization) (Jenkins 2011; VDI 3475 part 2 2005). The recommended WC at the beginning of the windrow pile is 50–60%, finishing the composting process with approximately 30% (Haug 1993; Misra et al. 2003; Sierra et al. 2017; VDI 3575 part 1 2003). In pile I, the WC at the beginning was around 60% and pile II was 66%. Since pile I experienced an early drying process, it was preferable to start with a higher WC value, which after 7 days, the windrow pile had reached the recommended value for the composting process. Pile III, on the other hand, kept a high WC value from the beginning until the last days of pile age. In the case of the coffee pulp, if its water content is comparatively low in porosity below 20–25% or above 60%, the aerobic process is stopped (Bidlingmaier 2003; Esquivel and Jiménez 2012; VDI 3475 part 3 2006). Above 60%, due to the dense structure of the coffee pulp (Table 1), it tends to keep high moisture content within if the material is not mixed or aerated regularly nor when there is no structural material in the windrow pile. The addition of structural material increases the volume of pore and therefore improves the exchange of water and air (Clausen 2015; Sánchez et al. 2015). Hence, structural material was considered in pile I and II to perform this work. Pile II, for 5 weeks, did not decrease the temperature profile, reaching the sanitation process during the last week, whereas pile III, after 5 weeks, decreased in temperature indicating less microbial activity. Therefore, coffee pulp needs proper WC, pH, oxygen, and porosity to reach a higher degradation. In addition to temperature measurement, the degradation of organic dry matter showed a rate approximately of 50% for pile I, 55% for pile II, and 34% for pile III.

On the other hand, detection of gaseous emissions is an essential measure for assessing the rotting process in terms of aerobic status and environmental relevance. On this basis, greenhouse gas and odor emissions can be reduced through optimized rotting management. The detection of gaseous emissions during the process of composting coffee by-products is one of the most important tools to meet the challenge of reducing GHG odor emissions in this sector. Gas concentrations act as indicators of a biological degradation and thus lead to optimization possibilities. Regarding the CH_4_ emission, previous studies show it is linked to the microorganisms’ activity and also connected to pH and temperature (Zhu-Barker et al. 2017). During the process, pile I and II follow the recommendations and moisture content profile of a proper composting process, while pile III maintains a high WC during the 35 days leading to constant CH_4_ emissions. It was seen that during the first week, pile III obtained the higher emissions, which might be linked to the amount of water content (85%), and lack of oxygen due to the compactness of coffee by-product.

During these measurements, the gas measured was CH_4_ since it is produced and oxidized during the degradation of organic matter with low O_2_ content and during the biological activity of the windrow pile (Phong and Cuhls 2016). CH_4_ formation is a product of anaerobic degradation forming organic acids as a result of methanogenesis (Hou et al. 2017; Msunar 2009). Since this gas is formed during endothermic reactions, and also when aerobic piles develope anaerobic zones inside of the pile during the composting process, this gas produce a reduction in the microorganism activity and as a consequence, CH_4_ emission (Phong and Cuhls 2016; VDI 3475 part 2 2005; VDI 3475 part 3 2006).

It is recommended in order to increase the porosity, oxygen, and decrease of emissions in the pile, the addition of green waste, branches or woodchips into the system, as well as the control of WC and temperature within the process. It was seen in pile III which possessed just coffee by-products, emissions up to 100 times higher than in pile I and II, where green waste and structural material was incorporated to give porosity to the windrow piles at the composting plant facility.

## Conclusions

Aerobic composting windrows were performed by using coffee by-products as a main component in a composting plant facility and were compared with the current treatment at the Mill, showing better profiles of temperature, pH, and WC when coffee by-products are mixed with green waste to form windrow piles.

Emission rates were determined and given in [g(m^2^)^−1^ h^−1^] based on the methodology described and compared with the emissions at the Mill in Costa Rica. CH_4_ emission rates were lower in pile I and II than in pile III where the highest emissions rates for 35 days found in pile I was 0.007 g(m^2^)^−1^ h^−1^, in pile II 0.006 g(m^2^)^−1^ h^−1^, while in pile III showed an emission of 3.1 g(m^2^)^−1^ h^−1^.

It was found that CH_4_ emissions could be avoided if the mixture and the formation of the windrow piles are done following the key parameter for composting, and therefore, the treatment at the Mill have the option to improve and to reduce the GHG emissions, giving the opportunity at the coffee sector during the management of the coffee by-products to improve the management of coffee by-products and to obtain a material with low emissions to be used afterwards in the coffee plantations as a fertilizer.
